# Scaling Up and Enhancing the Functionality of the Electronic Integrated Diseases Surveillance and Response System in Uganda, 2020-2022: Description of the Journey, Challenges, and Lessons Learned

**DOI:** 10.2196/59783

**Published:** 2025-04-14

**Authors:** Rodney Mugasha, Andrew Kwiringira, Vivian Ntono, Lydia Nakiire, Immaculate Ayebazibwe, Caroline Kyozira, Allan Niyonzima Muruta, Juliet Namugga Kasule, Dathan M Byonanebye, Judith Nanyondo, Richard Walwema, Francis Kakooza, Mohammed Lamorde

**Affiliations:** 1 Infectious Diseases Institute, College of Health Sciences, Makerere University, McKinnel Knowledge Center, 2 Hall Lane, Kampala, 10207, Uganda, 256 0776985284; 2 Health Information Systems Program Uganda, Kampala, Uganda; 3 Integrated Epidemiology, Surveillance, and Public Health Emergencies Department, Ministry of Health Uganda, Kampala, Uganda; 4 Division of Global Health Protection, Center for Global Health, Centers for Disease Control and Prevention, Kampala, Uganda

**Keywords:** electronic Integrated Disease Surveillance and Response, eIDSR, disease surveillance, training of trainers, Uganda, digital surveillance systems, health worker, eHealth, public health, digital health

## Abstract

In 2017, Uganda implemented an electronic Integrated Disease Surveillance and Response System (eIDSR) to improve data completeness and reporting timelines. However, the eIDSR system had limited functionality and was implemented on a small scale. The Ministry of Health, with support from the Infectious Disease Institute, Makerere University, and Health Information Systems Program Uganda, upgraded the system functionality and scaled up its implementation. This study describes the process and impact of upgrading eIDSR functionality and expanding its implementation across additional districts. The Ministry of Health, through its Integrated Epidemiology, Surveillance & Public Health Emergency Department, coordinated the implementation of the eIDSR. User requirements were identified through consultations with national surveillance stakeholders. The feedback informed the design and development of the upgraded eIDSR functionalities. The eIDSR rollout followed a consultative workshop to create awareness of the system among stakeholders. A curriculum was developed, and a national training of trainers was conducted. These trainers cascaded the training to the district health teams, who later cascaded the training to health workers. The training adopted an on-site training approach, where a group of national or district trainers would train new users at their desks. The eIDSR system was upgraded to the District Health Information Software 2 (DHIS2) 2.35 platform featuring faster reading and writing tracker data, handling over 100 concurrent users and enhanced case-based surveillance features on Android and web platforms. From October 2020 to September 2022, the eIDSR was rolled out in 68% (100/146) of districts. Additionally, the system permitted prompt reporting of signals of epidemic-prone diseases. Improving the functionality and the expanded geographical scope of the eIDSR system enhanced disease surveillance. Stakeholder commitment and leveraging existing structures will be needed to scale up eIDSR.

## Introduction

Uganda is highly susceptible to public health emergencies due to its proximity to the ecologically diverse and biologically rich tropical Congo Basin, recurrent epidemic-prone outbreaks, and refugee inflow [1]. In the last 5 years before 2024, Uganda has faced several significant outbreaks, including Ebola and Marburg virus diseases, cholera, measles, and Crimean-Congo hemorrhagic fever, highlighting the ongoing threat of infectious diseases [2]. Before 2000, surveillance systems were not robust enough to detect outbreaks early, leading to delayed responses and exacerbated public health impacts. For instance, the 2000 Ebola outbreak in Gulu, which resulted in 224 deaths, underscored the inadequacies in early warning and response mechanisms at the time [3].

**Figure 1.**
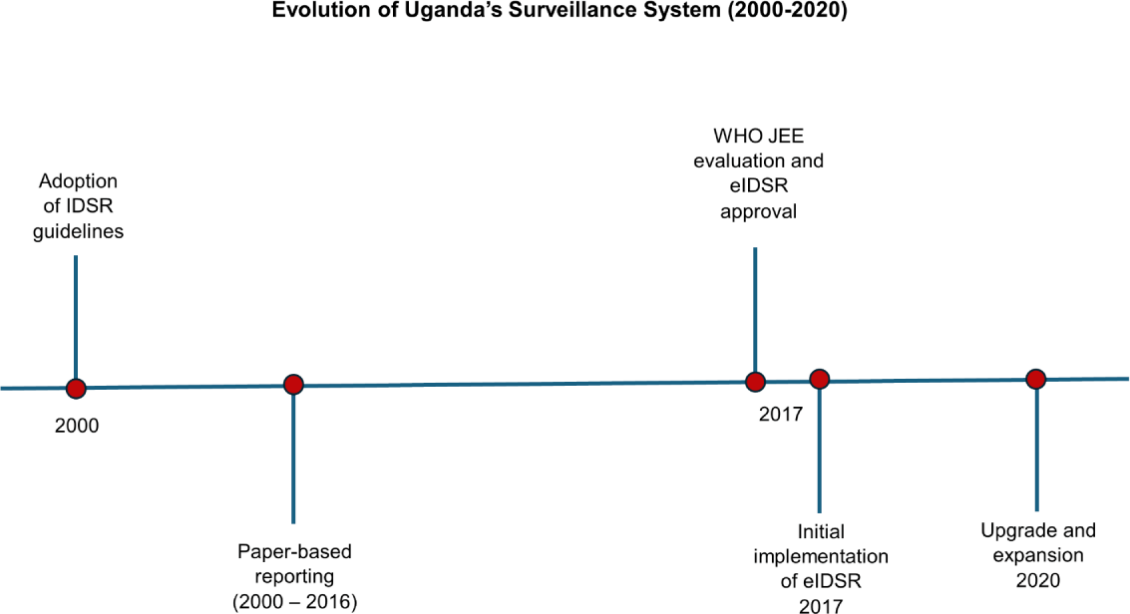
Road map of Uganda's surveillance system since 2000. eIDSR: electronic Integrated Disease Surveillance and Response; IDSR: Integrated Disease Surveillance and Response; WHO JEE: World Health Organization Joint External Evaluation.

Uganda embraced the Integrated Disease Surveillance and Response (IDSR) technical guidelines in 2000 [2]. However, the IDSR system was largely paper based. Reports from health facilities were transmitted physically to the district-by-district surveillance focal persons. The process of transporting paper-based reports often took several days to weeks, depending on the distance between the facilities and district offices, the availability of transportation, and other logistical challenges. This manual process led to delayed data transmission and response [2,4]. The World Health Organization (WHO) Joint External Evaluation conducted in 2017 highlighted the fragmentation and lack of coordination between human health, animal health, and laboratory data systems, which hindered effective surveillance of zoonotic diseases [3]. Additionally, the system faced challenges with data quality, including errors and inconsistencies due to manual data entry, as well as inadequate infrastructure, such as unreliable internet and computer systems, which further limited the effectiveness of the surveillance system [3]. In light of these findings, the Ministry of Health (MOH) approved the implementation of the electronic Integrated Disease Surveillance and Response (eIDSR) System in 2017 (Figure 1) to improve the timeliness of reporting; enhance data accuracy and quality; facilitate integration of data across human health, animal health, and laboratory sectors; and improve the capacity for data analysis and decision-making in public health interventions [5].

## The Electronic Integrated Disease Surveillance and Response System

The eIDSR system is based on the electronic application of the principles of the IDSR to facilitate early detection, investigation, and response to public health events [6]. The eIDSR was developed and customized on the District Health Information Software 2 (DHIS2) open-source software platform (version 2.35; Health Information Systems Program [HISP], University of Oslo) [7]. Unlike the conventional paper-based system used in health facilities, which requires data to be recaptured electronically at the district level, the eIDSR facilitates the instantaneous reporting of notifiable diseases through its SMS, Android, and web platforms [8]. The Android platform had an offline data entry functionality that allowed case-based notification and registration. The SMS platform facilitated immediate notification through a toll-free code currently used by the Uganda MOH. The application could also save data offline and automatically submit it as soon as an internet connection was available [9]. While improving paper-based reporting, the eIDSR system still faced challenges in ensuring that the data collected were complete and accurate. The eIDSR system facilitated case reporting through electronic case investigation forms for 9 out of 33 priority diseases and conditions. Inconsistencies in data entry and gaps in reporting affected the reliability of the information, which in turn impacted decision-making. The interface was not sufficiently user-friendly, which sometimes led to errors in data entry and delays in reporting. Moreover, the need for advanced data visualization tools made it difficult for public health officials to quickly interpret data trends and make informed decisions.

The enhancements and upgrades to the eIDSR system were therefore justified by the need to address these specific challenges. These upgrades included the addition of electronic case investigation forms for other priority diseases and advanced reporting tools to improve data timeliness and quality by streamlining the data entry process. New data visualization features were also added to enable public health officials to quickly interpret complex data through charts, graphs, and heat maps, facilitating more informed and timely public health actions. Additionally, enhancements were made to ensure better interoperability with other health information systems, thereby reducing data fragmentation and improving the overall efficiency of the surveillance system. The user interface was also redesigned to be more intuitive and user-friendly, reducing the likelihood of errors and encouraging more consistent use of the system by health care workers.

In October 2020, the Infectious Diseases Institute, Makerere University, in collaboration with the Uganda MOH and the HISP Uganda, upgraded the functionalities and scaled the eIDSR to 100 out of 146 districts. This study describes the process and impact of upgrading eIDSR functionality and expanding its implementation across additional districts.

## Methods

### Overview

A coordinated approach was used to systematically design the enhancement of functionalities and scale-up of the eIDSR. The process began with gathering user requirements from surveillance stakeholders. These requirements were then translated into technical upgrades. The MOH Integrated Epidemiology, Surveillance, and Public Health Emergencies (IES&PHE) Department led coordination. A cascade training model was then conducted to equip health care workers with the necessary skills.

### Coordination

The MOH, through its IES&PHE Department, coordinated the implementation of the eIDSR. The IES&PHE Department provided leadership and oversight throughout the implementation process. By coordinating the efforts of various stakeholders, the department ensured that the system was aligned with the country’s health priorities. The MOH coordinated the collection of user requirements to ensure that the eIDSR system addressed the specific needs of end-users, such as surveillance officers and health workers. Additionally, the MOH was responsible for convening national surveillance stakeholder meetings.

### User Requirements Gathering

User requirements were identified through consultations with national surveillance stakeholders, including the Ministry of Agriculture, Animal Industry, and Fisheries (MAAIF), implementing partners, as well as regional and district surveillance officers. The requirements were gathered on several aspects: system functionality, such as its ability to capture and transmit data in real-time; data quality, accuracy, completeness, and timeliness of reporting; user adoption; and operational challenges, such as technical issues and resource constraints. The feedback gathered was instrumental in informing the design and development of the upgraded eIDSR functionalities, which aimed to address gaps in the existing system.

### System Development and Enhancement

The technical team from the HISP Uganda then translated these requirements into operational enhancements. The eIDSR system was upgraded to the DHIS2 2.35 platform, featuring faster reading and writing tracker data and enhanced case-based surveillance features on Android and web platforms. Key changes included the integration of advanced reporting tools, real-time data visualization features, and enhanced interoperability with other health information systems, such as laboratory and animal health data systems. These upgrades were intended to streamline data collection allowing more concurrent users, improve accuracy, and enable more timely responses to public health threats (Figure 1).

National surveillance stakeholder engagement meetings were regularly convened to obtain user feedback and ensure ongoing coordination and harmonization of the eIDSR rollout. These meetings brought together representatives from the MOH, MAAIF, and other stakeholders to review system performance, address challenges, and plan the expansion of eIDSR to additional districts.

### Training

**Figure 2.**
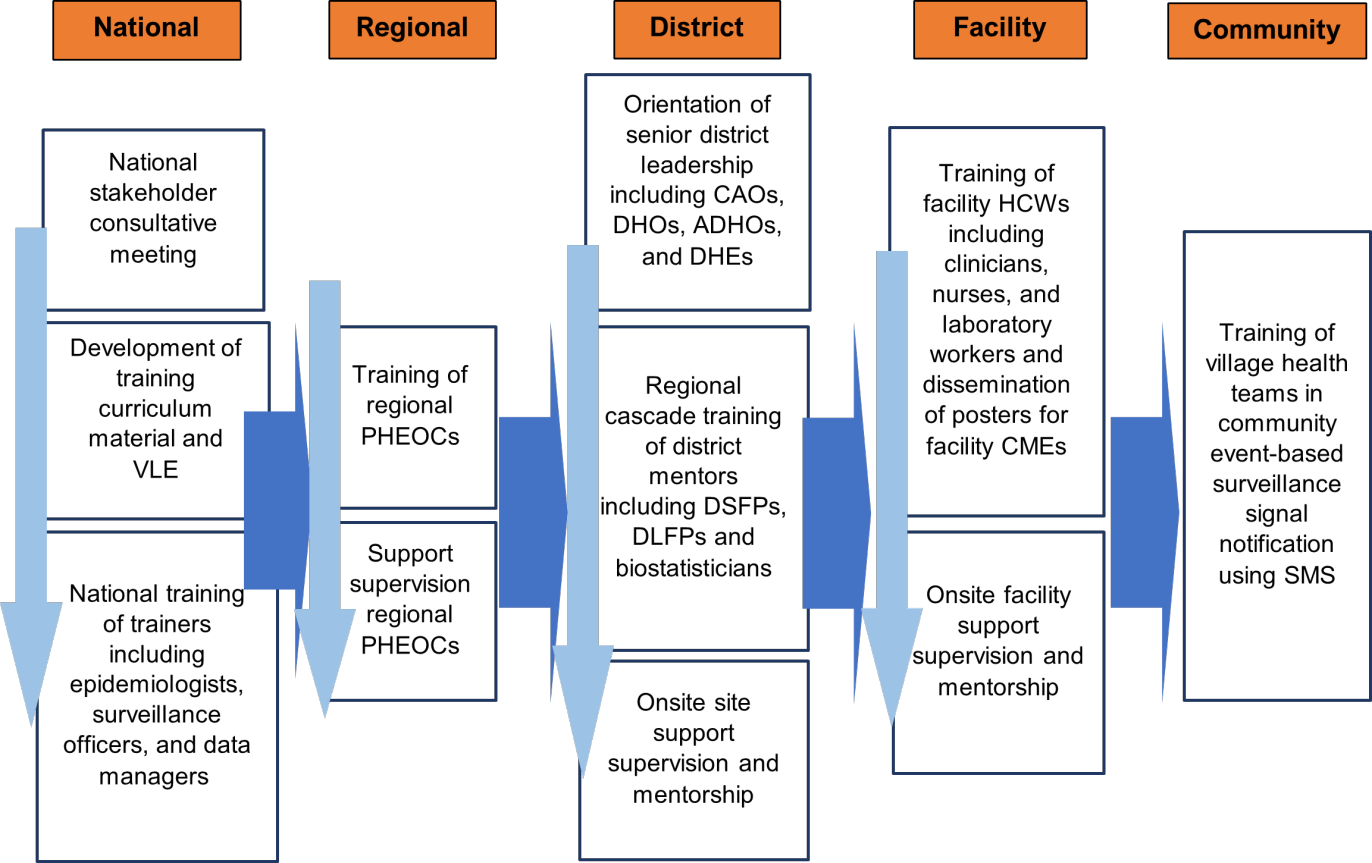
The electronic Integrated Disease Surveillance and Response (eIDSR) implementation approach in Uganda, 2020-2022. ADHO: assistant district health officer; CAO: chief administrative officer; CME: continuing medical education; DHE: district health educator; DHO: district health officer; DLFP: district laboratory focal person; DSFP: district surveillance focal person; HCW: health care worker; PHEOC: Public Health Emergency Operations Center; VLE: virtual learning environment.

An eIDSR curriculum was developed through a collaborative process involving key stakeholders from the MOH, implementing partners, and subject matter experts in epidemiology, surveillance, and health information systems. The development process began with a needs assessment to identify the specific skills and knowledge gaps among health workers at various levels of the surveillance system. The curriculum was further refined through a series of consultative workshops, where feedback was gathered from national and regional trainers, district health teams, and other stakeholders. This iterative process ensured that the training content was relevant, practical, and aligned with the MOH’s surveillance goals. Additionally, the curriculum incorporated IDSR best practices in disease surveillance and reporting, while being tailored to the local context of Uganda. Real-time simulations, case studies, and interactive sessions were added to make the training more engaging and to reinforce learning outcomes. The curriculum focused on alert notification, case notification, registration, and reporting using SMS, Android, and web platforms. The training curriculum also covered the fundamentals of surveillance, such as event-based surveillance and indicator-based surveillance, as well as priority diseases and events for immediate reporting. The rollout model adopted the MOH-recommended approach, where training was cascaded from the national to lower levels (Figure 2).

At the national level, national training of trainers was conducted to establish a cohort of national trainers to cascade training to the regional level. This training targeted epidemiologists; health information analysts; laboratory, clinical, monitoring, and evaluation specialists from the MOH; and implementing partners. At the regional level, the national trainers oriented the district health teams on the eIDSR. The district health team comprised of biostatisticians and the Health Management Information System focal persons, district surveillance focal persons, district laboratory focal persons, district veterinary officers, and health subdistrict surveillance focal persons.

At the district level, regional trainers trained clinicians, nurses, laboratory and health information assistants, and facility- and community-based health workers (ie, village health teams) at the facilities. Health workers were selected from high-volume facilities; regional and district hospitals; level 3 health centers that provide a broader range of services including outpatient and inpatient care, basic laboratory services, maternity services, and minor surgeries; and level 4 health centers that provide comprehensive care to larger areas with emergency obstetric care, basic surgeries, laboratory services, and treatment for common diseases. District surveillance focal persons that were trained were given tablets, monthly airtime, and internet data to support case registration and reporting of priority diseases. The trainers subsequently provided post-training mentorship and support supervision to subnational health teams through mentorship to ensure that skills gained during the training were sustained.

### System Upgrade and Stakeholder Engagement for eIDSR Enhancement

In 2020, the HISP Uganda developers improved the eIDSR system to the DHIS2 2.35 platform version following the user requirements gathering process. Improvements included the development of an additional 19 electronic case investigation forms for priority diseases, including rabies, anthrax, severe acute respiratory illnesses or influenza-like illnesses like COVID-19, and cholera. These diseases were identified due to their potential for outbreaks, high burden, and emerging threats. The existing eIDSR system lacked comprehensive tools to capture detailed case investigation data. The new forms were developed to address this gap by including essential variables such as exposure history, symptoms, and hospitalization details to enable effective disease tracking of case investigation details, exposure and travel history, symptoms, underlying conditions, and hospitalization.

The laboratory requests and results module were improved to capture laboratory requests for tests to be conducted on the specimen collected for each case. Variables captured include the identification, place, and time (date) of sample collection and the reference and final laboratory results. The improvements in the case outcome module also enabled more accurate tracking of health outcomes and final investigation results. The specimen tracking module was developed to link an existing system that facilitated tracking specimens across the laboratory hubs and laboratories until the completion of the case investigation. The module was developed to send an automated notification via email and SMS to the reference laboratory and the national disease surveillance team.

Additional upgrades to the system included enhanced data management and analytics capabilities that included the design of highly customizable forms to track individual-level data to facilitate case-based surveillance with data validation functionality for data quality assurance. Data analysis upgrades included robust charting capabilities to include epidemic curves to facilitate epidemiological data analysis. The upgrade included geographic information systems analysis using thematic mapping, allowing overlay of population, climate, and other layers using the Maps app and supporting geolocation of individual cases. In a bid to enhance situational awareness, a smart web-enabled television at the national level was procured to facilitate access to international news outlets and facilitate data visualization from the eIDSR.

### The eIDSR System Adoption and Use

**Figure 3.**
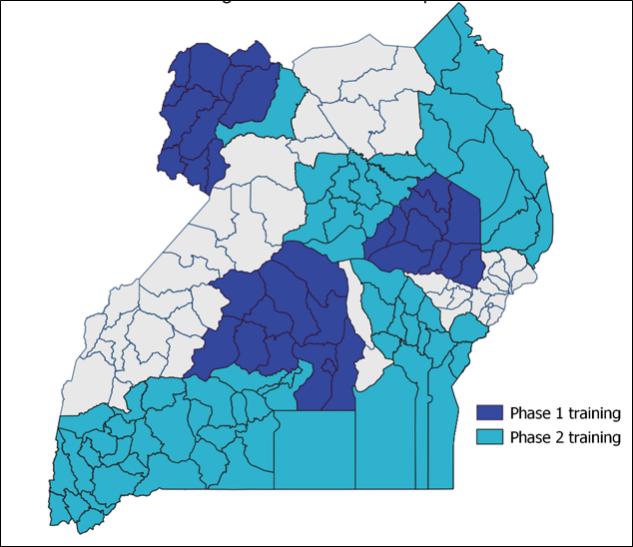
Coverage of the electronic Integrated Disease Surveillance and Response (eIDSR) phase 1 and phase 2 training in Uganda, 2020-2022.

During the 2 years of implementation (October 2020 and September 2021), the eIDSR was scaled up to 68% (100/146) of districts and cities (Figure 3). Thirty national trainers and 191 of the targeted 200 district-level mentors were trained. The district mentors subsequently trained and mentored 2409 of 3295 targeted facility-level health care workers from 536 health facilities. The project also trained another cohort of national-level surveillance officers and field epidemiologists from the Public Health Emergency Operations Center on system navigation, data extraction, analysis, and use for action.

Since its rollout, the eIDSR has continued to be an essential data source for disease surveillance to facilitate the detection and reporting of public health events countrywide. Several events were detected through the eIDSR from SMS messages sent from the community between 2020 and 2022 (Table 1).

**Table 1.** Outbreaks detected by the electronic Integrated Disease Surveillance and Response (eIDSR) in Uganda, 2020‐2022.

Disease outbreak or event	Districts that notified through the eIDSR	Date (year)
Anthrax	Madi-Okollo	2022
Undiagnosed deaths	Kyotera	2021
Rift Valley fever	Kagadi	2020
Anthrax	Bududa and Manafwa	2022
Crimean-Congo hemorrhagic fever	Kagadi	2020
Undiagnosed illness (skin rash)	Pakwach	2020
Rabies	West Nile region: Arua, Adjumani, Moyo, and Yumbe	2020‐2021
Suspected viral hemorrhagic fever: Rift Valley fever	Moyo, Obongi, Terego, Madi Okollo, and Yumbe	2020‐2021
Anthrax	Arua and Madi-Okollo	2021‐2022

### Challenges in Implementing the eIDSR

The eIDSR system in Uganda faces several challenges that could threaten the system’s sustainability. Data from system logs and user feedback indicate that insufficient technology infrastructure, including reliable internet connectivity and computer hardware, has led to delays in data submission from health care facilities.

Uganda faces significant challenges in its information and communication technology (ICT) infrastructure, impeding the successful implementation of the eIDSR in terms of real-time reporting. Inconsistent internet connectivity and limited access to computer hardware in some rural districts hindered the ability to report cases in real time similarly seen in other countries [10,11]. This was particularly problematic during peak times of disease outbreaks when timely data transmission was crucial for an effective response. It is challenging to operate ICT equipment due to limited access to broadband connectivity and electricity in some rural areas [12]. Another ICT-related challenge was the lack of smartphones among some district surveillance focal persons to register cases in the eIDSR mobile app. The lesson learned is that, for real-time reporting to be successful, ongoing investment in infrastructure, such as reliable internet and adequate hardware, is essential. Additionally, the importance of continuous monitoring and support to maintain data accuracy cannot be overstated.

Health care worker turnover and transfers have significantly impacted the successful implementation of the eIDSR. When trained staff leave, this creates skill shortages and disrupts continuity, causing a knowledge gap similarly seen in the first DHIS2 implementation [4]. This, coupled with the limited ICT skills among health care workers, especially in peripheral facilities, limits the adoption and effective use of the eIDSR. Continuous education and training of users is key to ensuring skill retention, and it is imperative to train many focal persons in each facility.

The inclusion of additional data variables for case investigations presented both an opportunity and a challenge. While the more detailed forms allowed for comprehensive data collection, health care workers found the increased data entry requirements burdensome, particularly in high-volume settings. This sometimes led to incomplete case investigations and delays in submitting reports. To address this, additional training and on-the-job support were provided, emphasizing the importance of thorough and timely data entry. The lesson learned is that there is a need to balance data comprehensiveness with the workload of health care workers to ensure that the system remains user-friendly and efficient.

### Opportunities

Despite these challenges, the upgrades to the eIDSR system have provided several opportunities for enhancing disease surveillance and response. The improved system functionality, combined with the lessons learned from the challenges faced, have paved the way for future improvements. For instance, the introduction of mobile data collection tools and offline data entry options could further enhance real-time reporting capabilities, especially in remote areas. Additionally, increased stakeholder engagement and continuous feedback from users will be critical in refining the system and ensuring its long-term sustainability. The pivotal role played by the MOH throughout the implementation process, coupled with the active involvement of district health team trainers, creates a strong possibility of the government taking ownership of eIDSR implementation, independent of external project support.

## Discussion

We successfully upgraded eIDSR system functionality and scaled up to more than half of the districts in the country within 2 years of implementation. We also established a cohort of surveillance staff at national and subnational levels who could train health workers across all levels of health care. The implementation of eIDSR was one of the key priority actions in real-time surveillance to address gaps that were recommended from the last WHO Joint External Evaluation conducted in 2017.

The WHO IDSR framework, which strongly emphasizes the necessity for electronic surveillance tools to allow a wide range of crucial surveillance functions, served as a guide for improving the functionality of the eIDSR system [6]. These functions encompass alert notification, case investigation and registration, data quality assurance, real-time reporting, and monitoring and evaluation [9]. The expanded goal of enhancing the national electronic surveillance system has the potential to benefit significantly from the enhanced functionality of the eIDSR. This broader system intends to seamlessly integrate laboratory tests, notification processes, emergency responses, and reporting mechanisms with indicator-based surveillance, event-based surveillance, and case-based surveillance data across the human health sector. Notably, the eIDSR adheres to open standards and was carefully created on the DHIS2 platform, enabling smooth interchange and connectivity with other software applications and data sources [12]. This strategic alignment puts Uganda on a trajectory for developing interoperability with numerous electronic surveillance systems across multiple industries, in addition to laboratory systems.

A crucial element of the successful rollout of the eIDSR system was the establishment of the curriculum and training of health personnel. Learning is improved by creating a thorough curriculum that incorporates on-site mentoring improvement [13,14]. It was crucial to have onsite supervision and experienced national trainers with knowledge of the eIDSR, public health, and disease monitoring. Maintaining competency requires regular monitoring and evaluation, which includes refresher training. Documenting training records maintains accountability. Sustainability is promoted by incorporating eIDSR training into already-existing health care training programs, and community involvement increases understanding of the system’s significance and the responsibilities that health care professionals play in disease monitoring, ultimately improving public health outcomes.

The commitment of the MOH and existing administrative structures provided an opportunity to implement the eIDSR. For example, the division of health information in the MOH is mandated to coordinate initiatives to digitize the health infrastructure [15]. The IES&PHE Department coordinates surveillance and response to public health emergencies. However, several factors documented by unpublished support supervision reports could threaten the sustainability of the system even after enhancement of the system; these include workforce challenges, such as weak ICT infrastructure at the district and health facility level and insufficient funding at the district to conduct surveillance activities. Additionally, Uganda does not have in place interoperable and interconnected electronic reporting for animal health [3]. Data use is lacking as we wait for outbreaks to act rather than use available data to predict specific disease outbreaks, and analysis also remains a challenge.

There are potential limitations to this project. First, the analysis was not intended to be a formal review of the eIDSR platform; we did not assess how the system performed. Thus, additional research is needed to examine to what extent the intervention contributed to improved reporting rates. Secondly, this project did not train village health teams due to inadequate funding. Our project did not give phones or tablets to facility health workers; therefore, we cannot make definitive claims about the effect of implementing the eIDSR on improving case reporting rates.

## Conclusions

Improving the functionality and expanding the eIDSR system’s geographical scope improved the efficiency of disease surveillance. The system’s enhanced features, such as advanced reporting tools, improved data timeliness by streamlining the data entry process, and visualization features enabled quick interpretation of complex data, facilitating decision-making.

The wider geographical reach signifies a successful initial scale-up. To ensure the sustained success and scale of the eIDSR system, future programs should facilitate stakeholder commitment and leverage existing structures. Engaging internal and external stakeholders is crucial for securing long-term support, commitment, and endorsement at the national and subnational levels.

## Abbreviations

DHIS2: District Health Information Software 2
eIDSR: electronic Integrated Disease Surveillance and Response
HISP: Health Information Systems Program
ICT: information and communication technology
IDSR: Integrated Disease Surveillance and Response
IES&PHE: Integrated Epidemiology, Surveillance, and Public Health Emergencies
MAAIF: Ministryof Agriculture, Animal Industry, and Fisheries
MOH: Ministry of Health
WHO: World Health Organization
